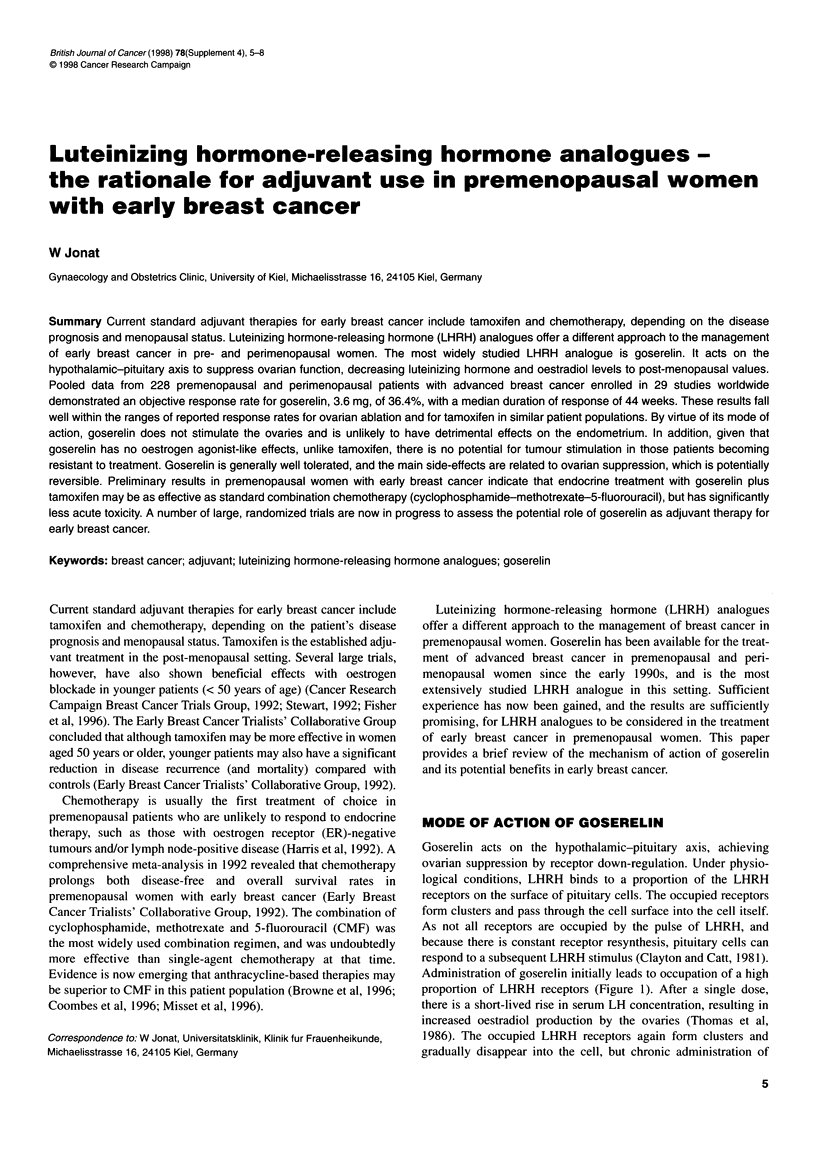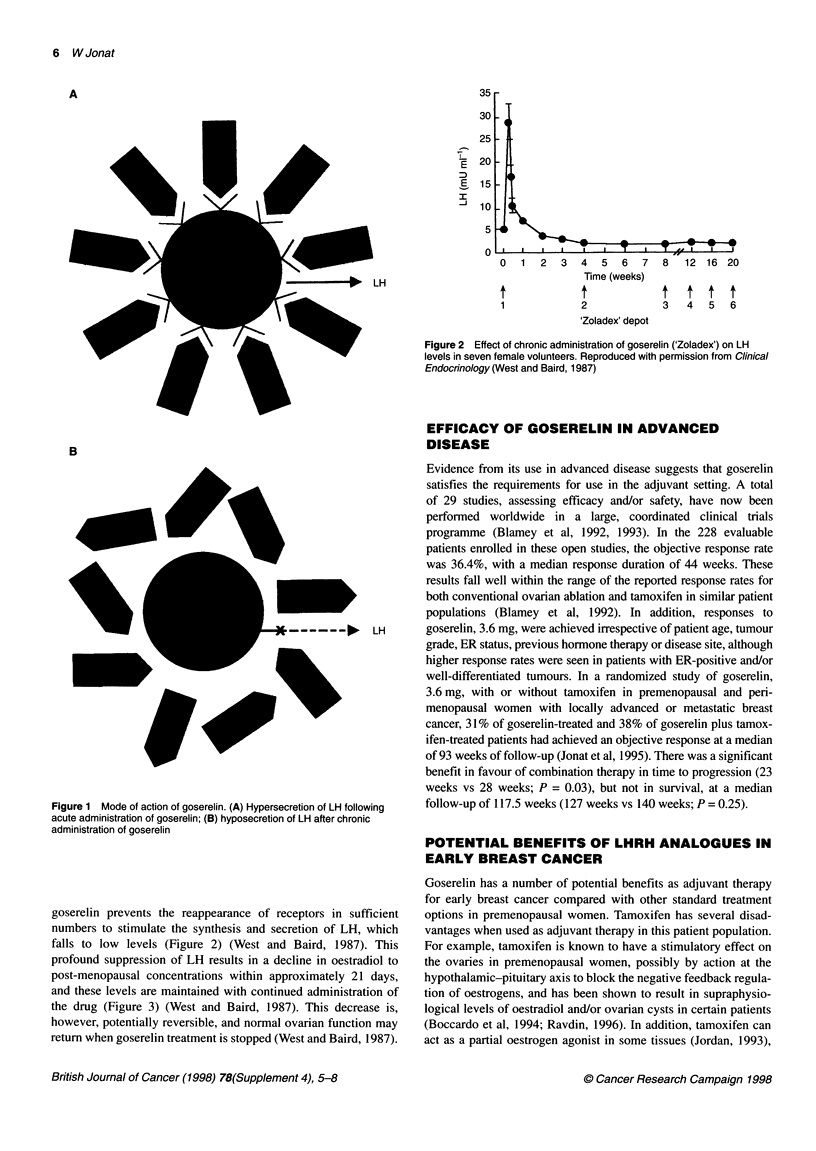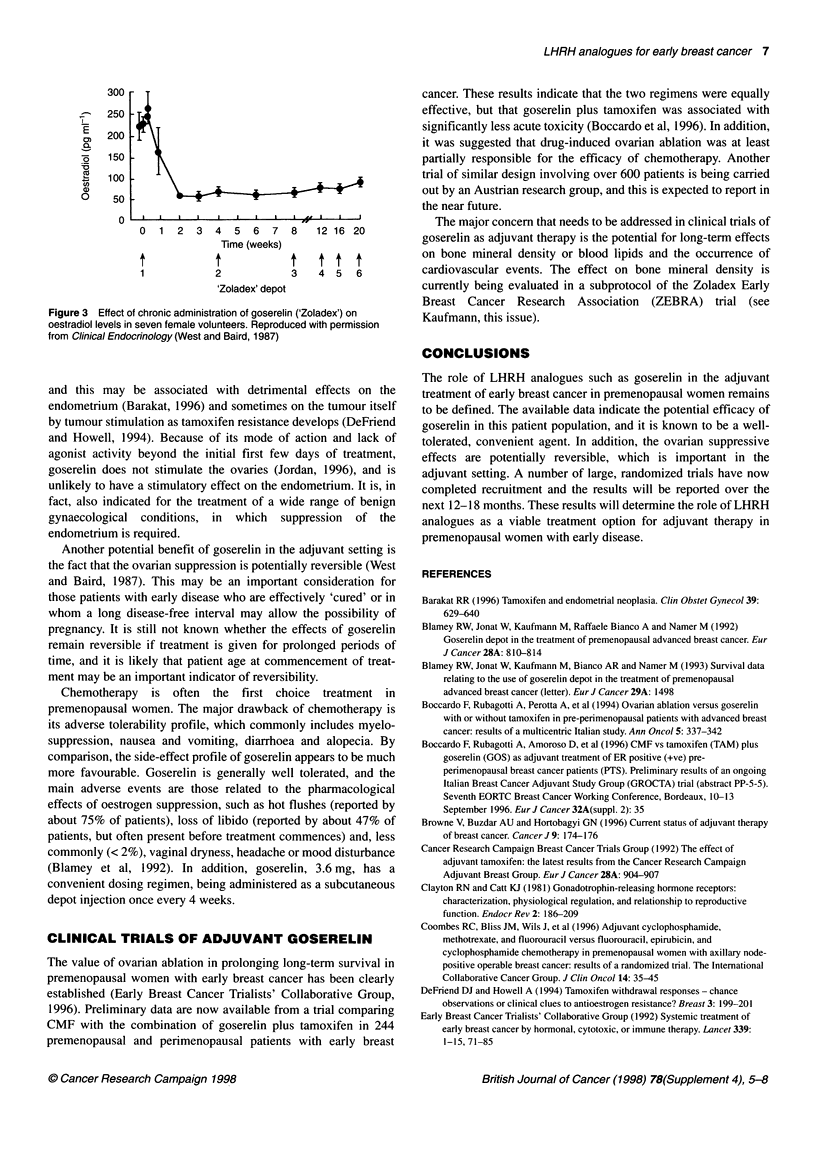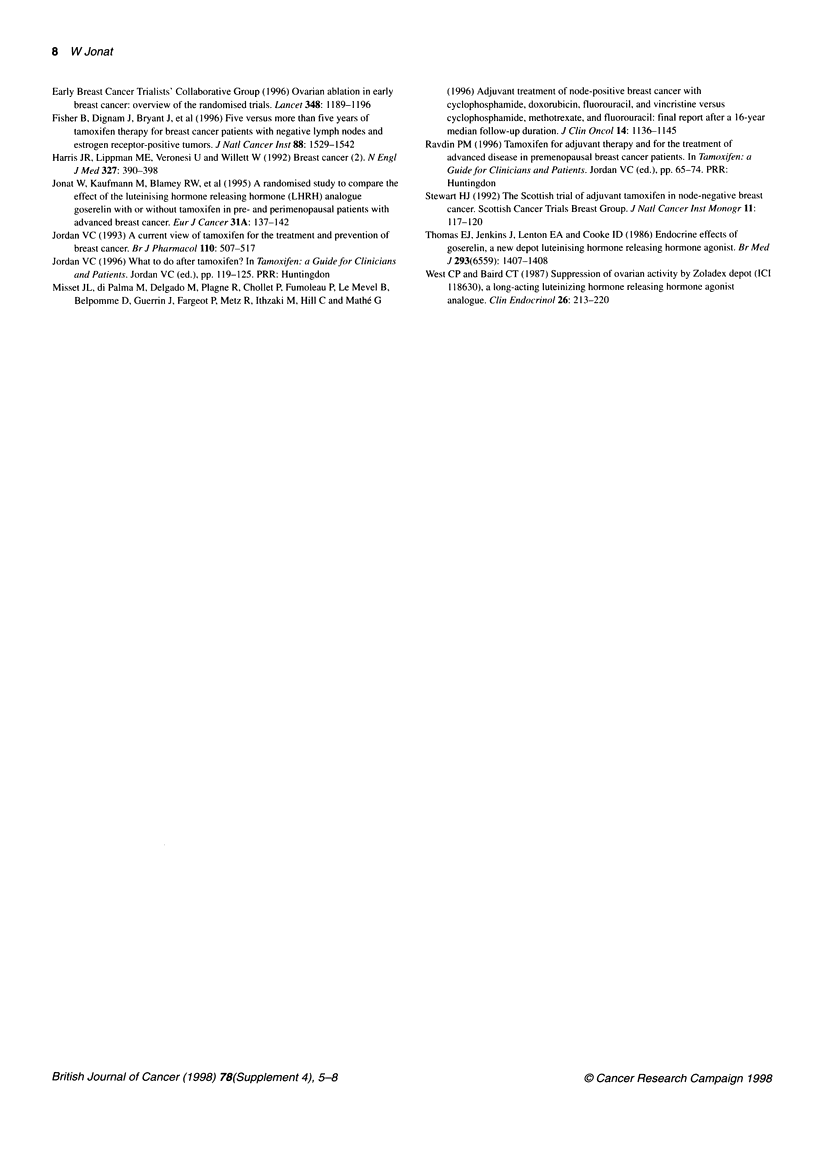# Luteinizing hormone-releasing hormone analogues--the rationale for adjuvant use in premenopausal women with early breast cancer.

**DOI:** 10.1038/bjc.1998.754

**Published:** 1998-09

**Authors:** W. Jonat

**Affiliations:** Gynaecology and Obstetrics Clinic, University of Kiel, Germany.

## Abstract

Current standard adjuvant therapies for early breast cancer include tamoxifen and chemotherapy, depending on the disease prognosis and menopausal status. Luteinizing hormone-releasing hormone (LHRH) analogues offer a different approach to the management of early breast cancer in pre- and perimenopausal women. The most widely studied LHRH analogue is goserelin. It acts on the hypothalamic-pituitary axis to suppress ovarian function, decreasing luteinizing hormone and oestradiol levels to post-menopausal values. Pooled data from 228 premenopausal and perimenopausal patients with advanced breast cancer enrolled in 29 studies worldwide demonstrated an objective response rate for goserelin, 3.6 mg, of 36.4%, with a median duration of response of 44 weeks. These results fall well within the ranges of reported response rates for ovarian ablation and for tamoxifen in similar patient populations. By virtue of its mode of action, goserelin does not stimulate the ovaries and is unlikely to have detrimental effects on the endometrium. In addition, given that goserelin has no oestrogen agonist-like effects, unlike tamoxifen, there is no potential for tumour stimulation in those patients becoming resistant to treatment. Goserelin is generally well tolerated, and the main side-effects are related to ovarian suppression, which is potentially reversible. Preliminary results in premenopausal women with early breast cancer indicate that endocrine treatment with goserelin plus tamoxifen may be as effective as standard combination chemotherapy (cyclophosphamide-methotrexate-5-fluorouracil), but has significantly less acute toxicity. A number of large, randomized trials are now in progress to assess the potential role of goserelin as adjuvant therapy for early breast cancer.


British Journal of Cancer (1998) 78(Supplement 4), 5–8
© 1998 Cancer Research Campaign

### Luteinizing hormone-releasing hormone analogues – the rationale for adjuvant use in premenopausal women with early breast cancer

**W Jonat**

Gynaecology and Obstetrics Clinic, University of Kiel, Michaelisstrasse 16, 24105 Kiel, Germany

**Summary** Current standard adjuvant therapies for early breast cancer include tamoxifen and chemotherapy, depending on the disease prognosis and menopausal status. Luteinizing hormone-releasing hormone (LHRH) analogues offer a different approach to the management of early breast cancer in pre- and perimenopausal women. The most widely studied LHRH analogue is goserelin. It acts on the hypothalamic–pituitary axis to suppress ovarian function, decreasing luteinizing hormone and oestradiol levels to post-menopausal values. Pooled data from 228 premenopausal and perimenopausal patients with advanced breast cancer enrolled in 29 studies worldwide demonstrated an objective response rate for goserelin, 3.6 mg, of 36.4%, with a median duration of response of 44 weeks. These results fall well within the ranges of reported response rates for ovarian ablation and for tamoxifen in similar patient populations. By virtue of its mode of action, goserelin does not stimulate the ovaries and is unlikely to have detrimental effects on the endometrium. In addition, given that goserelin has no oestrogen agonist-like effects, unlike tamoxifen, there is no potential for tumour stimulation in those patients becoming resistant to treatment. Goserelin is generally well tolerated, and the main side-effects are related to ovarian suppression, which is potentially reversible. Preliminary results in premenopausal women with early breast cancer indicate that endocrine treatment with goserelin plus tamoxifen may be as effective as standard combination chemotherapy (cyclophosphamide–methotrexate–5-fluorouracil), but has significantly less acute toxicity. A number of large, randomized trials are now in progress to assess the potential role of goserelin as adjuvant therapy for early breast cancer.

**Keywords:** breast cancer; adjuvant; luteinizing hormone-releasing hormone analogues; goserelin

Current standard adjuvant therapies for early breast cancer include tamoxifen and chemotherapy, depending on the patient's disease prognosis and menopausal status. Tamoxifen is the established adjuvant treatment in the post-menopausal setting. Several large trials, however, have also shown beneficial effects with oestrogen blockade in younger patients (< 50 years of age) (Cancer Research Campaign Breast Cancer Trials Group, 1992; Stewart, 1992; Fisher et al, 1996). The Early Breast Cancer Trialists' Collaborative Group concluded that although tamoxifen may be more effective in women aged 50 years or older, younger patients may also have a significant reduction in disease recurrence (and mortality) compared with controls (Early Breast Cancer Trialists' Collaborative Group, 1992).

Chemotherapy is usually the first treatment of choice in premenopausal patients who are unlikely to respond to endocrine therapy, such as those with oestrogen receptor (ER)-negative tumours and/or lymph node-positive disease (Harris et al, 1992). A comprehensive meta-analysis in 1992 revealed that chemotherapy prolongs both disease-free and overall survival rates in premenopausal women with early breast cancer (Early Breast Cancer Trialists' Collaborative Group, 1992). The combination of cyclophosphamide, methotrexate and 5-fluorouracil (CMF) was the most widely used combination regimen, and was undoubtedly more effective than single-agent chemotherapy at that time. Evidence is now emerging that anthracycline-based therapies may be superior to CMF in this patient population (Browne et al, 1996; Coombes et al, 1996; Misset et al, 1996).

*Correspondence to:* W Jonat, Universitatsklinik, Klinik fur Frauenheikunde, Michaelisstrasse 16, 24105 Kiel, Germany

Luteinizing hormone-releasing hormone (LHRH) analogues offer a different approach to the management of breast cancer in premenopausal women. Goserelin has been available for the treatment of advanced breast cancer in premenopausal and perimenopausal women since the early 1990s, and is the most extensively studied LHRH analogue in this setting. Sufficient experience has now been gained, and the results are sufficiently promising, for LHRH analogues to be considered in the treatment of early breast cancer in premenopausal women. This paper provides a brief review of the mechanism of action of goserelin and its potential benefits in early breast cancer.

### MODE OF ACTION OF GOSERELIN

Goserelin acts on the hypothalamic–pituitary axis, achieving ovarian suppression by receptor down-regulation. Under physiological conditions, LHRH binds to a proportion of the LHRH receptors on the surface of pituitary cells. The occupied receptors form clusters and pass through the cell surface into the cell itself. As not all receptors are occupied by the pulse of LHRH, and because there is constant receptor resynthesis, pituitary cells can respond to a subsequent LHRH stimulus (Clayton and Catt, 1981). Administration of goserelin initially leads to occupation of a high proportion of LHRH receptors (Figure 1). After a single dose, there is a short-lived rise in serum LH concentration, resulting in increased oestradiol production by the ovaries (Thomas et al, 1986). The occupied LHRH receptors again form clusters and gradually disappear into the cell, but chronic administration of

A

B

**Figure 1** Mode of action of goserelin. (**A**) Hypersecretion of LH following acute administration of goserelin; (**B**) hyposecretion of LH after chronic administration of goserelin goserelin prevents the reappearance of receptors in sufficient numbers to stimulate the synthesis and secretion of LH, which falls to low levels (Figure 2) (West and Baird, 1987). This profound suppression of LH results in a decline in oestradiol to post-menopausal concentrations within approximately 21 days, and these levels are maintained with continued administration of the drug (Figure 3) (West and Baird, 1987). This decrease is, however, potentially reversible, and normal ovarian function may return when goserelin treatment is stopped (West and Baird, 1987).

**Figure 2** Effect of chronic administration of goserelin ('Zoladex') on LH levels in seven female volunteers. Reproduced with permission from *Clinical Endocrinology* (West and Baird, 1987)

### EFFICACY OF GOSERELIN IN ADVANCED DISEASE

Evidence from its use in advanced disease suggests that goserelin satisfies the requirements for use in the adjuvant setting. A total of 29 studies, assessing efficacy and/or safety, have now been performed worldwide in a large, coordinated clinical trials programme (Blamey et al, 1992, 1993). In the 228 evaluable patients enrolled in these open studies, the objective response rate was 36.4%, with a median response duration of 44 weeks. These results fall well within the range of the reported response rates for both conventional ovarian ablation and tamoxifen in similar patient populations (Blamey et al, 1992). In addition, responses to goserelin, 3.6 mg, were achieved irrespective of patient age, tumour grade, ER status, previous hormone therapy or disease site, although higher response rates were seen in patients with ER-positive and/or well-differentiated tumours. In a randomized study of goserelin, 3.6 mg, with or without tamoxifen in premenopausal and peri-menopausal women with locally advanced or metastatic breast cancer, 31% of goserelin-treated and 38% of goserelin plus tamoxifen-treated patients had achieved an objective response at a median of 93 weeks of follow-up (Jonat et al, 1995). There was a significant benefit in favour of combination therapy in time to progression (23 weeks vs 28 weeks; $P = 0.03$), but not in survival, at a median follow-up of 117.5 weeks (127 weeks vs 140 weeks; $P = 0.25$).

### POTENTIAL BENEFITS OF LHRH ANALOGUES IN EARLY BREAST CANCER

Goserelin has a number of potential benefits as adjuvant therapy for early breast cancer compared with other standard treatment options in premenopausal women. Tamoxifen has several disadvantages when used as adjuvant therapy in this patient population. For example, tamoxifen is known to have a stimulatory effect on the ovaries in premenopausal women, possibly by action at the hypothalamic–pituitary axis to block the negative feedback regulation of oestrogens, and has been shown to result in supraphysiological levels of oestradiol and/or ovarian cysts in certain patients (Boccardo et al, 1994; Ravdin, 1996). In addition, tamoxifen can act as a partial oestrogen agonist in some tissues (Jordan, 1993),

 © Cancer Research Campaign 1998

**Figure 3** Effect of chronic administration of goserelin ('Zoladex') on oestradiol levels in seven female volunteers. Reproduced with permission from *Clinical Endocrinology* (West and Baird, 1987)

and this may be associated with detrimental effects on the endometrium (Barakat, 1996) and sometimes on the tumour itself by tumour stimulation as tamoxifen resistance develops (DeFriend and Howell, 1994). Because of its mode of action and lack of agonist activity beyond the initial first few days of treatment, goserelin does not stimulate the ovaries (Jordan, 1996), and is unlikely to have a stimulatory effect on the endometrium. It is, in fact, also indicated for the treatment of a wide range of benign gynaecological conditions, in which suppression of the endometrium is required.

Another potential benefit of goserelin in the adjuvant setting is the fact that the ovarian suppression is potentially reversible (West and Baird, 1987). This may be an important consideration for those patients with early disease who are effectively 'cured' or in whom a long disease-free interval may allow the possibility of pregnancy. It is still not known whether the effects of goserelin remain reversible if treatment is given for prolonged periods of time, and it is likely that patient age at commencement of treatment may be an important indicator of reversibility.

Chemotherapy is often the first choice treatment in premenopausal women. The major drawback of chemotherapy is its adverse tolerability profile, which commonly includes myelosuppression, nausea and vomiting, diarrhoea and alopecia. By comparison, the side-effect profile of goserelin appears to be much more favourable. Goserelin is generally well tolerated, and the main adverse events are those related to the pharmacological effects of oestrogen suppression, such as hot flushes (reported by about 75% of patients), loss of libido (reported by about 47% of patients, but often present before treatment commences) and, less commonly (< 2%), vaginal dryness, headache or mood disturbance (Blamey et al, 1992). In addition, goserelin, 3.6 mg, has a convenient dosing regimen, being administered as a subcutaneous depot injection once every 4 weeks.

### CLINICAL TRIALS OF ADJUVANT GOSERELIN

The value of ovarian ablation in prolonging long-term survival in premenopausal women with early breast cancer has been clearly established (Early Breast Cancer Trialists' Collaborative Group, 1996). Preliminary data are now available from a trial comparing CMF with the combination of goserelin plus tamoxifen in 244 premenopausal and perimenopausal patients with early breast cancer. These results indicate that the two regimens were equally effective, but that goserelin plus tamoxifen was associated with significantly less acute toxicity (Boccardo et al, 1996). In addition, it was suggested that drug-induced ovarian ablation was at least partially responsible for the efficacy of chemotherapy. Another trial of similar design involving over 600 patients is being carried out by an Austrian research group, and this is expected to report in the near future.

The major concern that needs to be addressed in clinical trials of goserelin as adjuvant therapy is the potential for long-term effects on bone mineral density or blood lipids and the occurrence of cardiovascular events. The effect on bone mineral density is currently being evaluated in a subprotocol of the Zoladex Early Breast Cancer Research Association (ZEBRA) trial (see Kaufmann, this issue).

### CONCLUSIONS

The role of LHRH analogues such as goserelin in the adjuvant treatment of early breast cancer in premenopausal women remains to be defined. The available data indicate the potential efficacy of goserelin in this patient population, and it is known to be a well-tolerated, convenient agent. In addition, the ovarian suppressive effects are potentially reversible, which is important in the adjuvant setting. A number of large, randomized trials have now completed recruitment and the results will be reported over the next 12–18 months. These results will determine the role of LHRH analogues as a viable treatment option for adjuvant therapy in premenopausal women with early disease.

### REFERENCES

Barakat RR (1996) Tamoxifen and endometrial neoplasia. *Clin Obstet Gynecol* **39**: 629–640

Blamey RW, Jonat W, Kaufmann M, Raffaele Bianco A and Namer M (1992) Goserelin depot in the treatment of premenopausal advanced breast cancer. *Eur J Cancer* **28A**: 810–814

Blamey RW, Jonat W, Kaufmann M, Bianco AR and Namer M (1993) Survival data relating to the use of goserelin depot in the treatment of premenopausal advanced breast cancer (letter). *Eur J Cancer* **29A**: 1498

Boccardo F, Rubagotti A, Perotta A, et al (1994) Ovarian ablation versus goserelin with or without tamoxifen in pre-perimenopausal patients with advanced breast cancer: results of a multicentric Italian study. *Ann Oncol* **5**: 337–342

Boccardo F, Rubagotti A, Amoroso D, et al (1996) CMF vs tamoxifen (TAM) plus goserelin (GOS) as adjuvant treatment of ER positive (+ve) pre-perimenopausal breast cancer patients (PTS). Preliminary results of an ongoing Italian Breast Cancer Adjuvant Study Group (GROCTA) trial (abstract PP-5-5). Seventh EORTC Breast Cancer Working Conference, Bordeaux, 10–13 September 1996. *Eur J Cancer* **32A**(suppl. 2): 35

Browne V, Buzdar AU and Hortobagyi GN (1996) Current status of adjuvant therapy of breast cancer. *Cancer J* **9**: 174–176

Cancer Research Campaign Breast Cancer Trials Group (1992) The effect of adjuvant tamoxifen: the latest results from the Cancer Research Campaign Adjuvant Breast Group. *Eur J Cancer* **28A**: 904–907

Clayton RN and Catt KJ (1981) Gonadotrophin-releasing hormone receptors: characterization, physiological regulation, and relationship to reproductive function. *Endocr Rev* **2**: 186–209

Coombes RC, Bliss JM, Wils J, et al (1996) Adjuvant cyclophosphamide, methotrexate, and fluorouracil versus fluorouracil, epirubicin, and cyclophosphamide chemotherapy in premenopausal women with axillary node-positive operable breast cancer: results of a randomized trial. The International Collaborative Cancer Group. *J Clin Oncol* **14**: 35–45

DeFriend DJ and Howell A (1994) Tamoxifen withdrawal responses – chance observations or clinical clues to antioestrogen resistance? *Breast* **3**: 199–201

Early Breast Cancer Trialists' Collaborative Group (1992) Systemic treatment of early breast cancer by hormonal, cytotoxic, or immune therapy. *Lancet* **339**: 1–15, 71–85

© Cancer Research Campaign 1998

Early Breast Cancer Trialists' Collaborative Group (1996) Ovarian ablation in early breast cancer: overview of the randomised trials. *Lancet* **348**: 1189–1196

Fisher B, Dignam J, Bryant J, et al (1996) Five versus more than five years of tamoxifen therapy for breast cancer patients with negative lymph nodes and estrogen receptor-positive tumors. *J Natl Cancer Inst* **88**: 1529–1542

Harris JR, Lippman ME, Veronesi U and Willett W (1992) Breast cancer (2). *N Engl J Med* **327**: 390–398

Jonat W, Kaufmann M, Blamey RW, et al (1995) A randomised study to compare the effect of the luteinising hormone releasing hormone (LHRH) analogue goserelin with or without tamoxifen in pre- and perimenopausal patients with advanced breast cancer. *Eur J Cancer* **31A**: 137–142

Jordan VC (1993) A current view of tamoxifen for the treatment and prevention of breast cancer. *Br J Pharmacol* **110**: 507–517

Jordan VC (1996) What to do after tamoxifen? In *Tamoxifen: a Guide for Clinicians and Patients*. Jordan VC (ed.), pp. 119–125. PRR: Huntingdon

Misset JL, di Palma M, Delgado M, Plagne R, Chollet P, Fumoleau P, Le Mevel B, Belpomme D, Guerrin J, Fargeot P, Metz R, Ithzaki M, Hill C and Mathé G (1996) Adjuvant treatment of node-positive breast cancer with cyclophosphamide, doxorubicin, fluorouracil, and vincristine versus cyclophosphamide, methotrexate, and fluorouracil: final report after a 16-year median follow-up duration. *J Clin Oncol* **14**: 1136–1145

Ravdin PM (1996) Tamoxifen for adjuvant therapy and for the treatment of advanced disease in premenopausal breast cancer patients. In *Tamoxifen: a Guide for Clinicians and Patients*. Jordan VC (ed.), pp. 65–74. PRR: Huntingdon

Stewart HJ (1992) The Scottish trial of adjuvant tamoxifen in node-negative breast cancer. Scottish Cancer Trials Breast Group. *J Natl Cancer Inst Monogr* **11**: 117–120

Thomas EJ, Jenkins J, Lenton EA and Cooke ID (1986) Endocrine effects of goserelin, a new depot luteinising hormone releasing hormone agonist. *Br Med J* **293**(6559): 1407–1408

West CP and Baird CT (1987) Suppression of ovarian activity by Zoladex depot (ICI 118630), a long-acting luteinizing hormone releasing hormone agonist analogue. *Clin Endocrinol* **26**: 213–220

*© Cancer Research Campaign 1998*